# Nanosurveyor: a framework for real-time data processing

**DOI:** 10.1186/s40679-017-0039-0

**Published:** 2017-01-31

**Authors:** Benedikt J. Daurer, Hari Krishnan, Talita Perciano, Filipe R. N. C. Maia, David A. Shapiro, James A. Sethian, Stefano Marchesini

**Affiliations:** 1 Laboratory of Molecular Biophysics, Department of Cell and Molecular Biology, Uppsala University, Uppsala, Sweden; 2 Computational Research Division, Lawrence Berkeley National Laboratory, Berkeley, CA USA; 3 NERSC, Lawrence Berkeley National Laboratory, Berkeley, CA USA; 4 Advanced Light Source, Lawrence Berkeley National Laboratory, Berkeley, CA USA; 5 Department of Mathematics, University of California, Berkeley, Berkeley, CA USA

**Keywords:** Streaming, Ptychography

## Abstract

**Background:**

The ever improving brightness of accelerator based sources is enabling novel observations and discoveries with faster frame rates, larger fields of view, higher resolution, and higher dimensionality.

**Results:**

Here we present an integrated software/algorithmic framework designed to capitalize on high-throughput experiments through efficient kernels, load-balanced workflows, which are scalable in design. We describe the streamlined processing pipeline of ptychography data analysis.

**Conclusions:**

The pipeline provides throughput, compression, and resolution as well as rapid feedback to the microscope operators.

## Background

When new drugs are synthesized [1], dust particles are brought back from space [2], or new superconductors are discovered [3], a variety of sophisticated X-ray microscopes, spectrometers, and scattering instruments are often summoned to characterize their structure and properties. High-resolution and hyperspectral X-ray imaging, scattering and tomography instruments at modern synchrotrons are among the workhorses of modern discovery to study nano-materials and characterize chemical interactions or electronic properties at their interfaces.

A new generation of microscopes are being pioneered, commissioned, and planned at several US Department of Energy (DOE) user facilities [4–6] and elsewhere to achieve superior resolution and contrast in three dimensions, encompassing a macroscopic field of view and chemical or magnetic sensitivity, by coupling together the brightest sources of tunable X-rays, nanometer positioning, nanofocusing lenses, and faster detectors. Existing soft X-ray detector technology in use at the Advanced Light Source (ALS) for example generates 350 MBytes/s per instrument [7]; commercial detectors for hard X-rays can record 6 GB/s or raw data per detector  [8, 9], and a synchrotron light source can support 40 or more experiments simultaneously 24 hours a day. Accelerator technologies such as multi-bend achromat [10] are poised to increase brightness by two orders of magnitude around the globe  [11, 12]. Next generation microscopes may exploit multi-color sources, increased detector parallelism, increased frame rate, or stroboscopic structured illumination to extract higher-dimensional, higher resolution, higher frame-rate characterization of a specimen. There is a need for reducing data into meaningful images as rapidly as it is acquired, using low-cost algorithms and computational resources.

Modern synchrotron experiments often have quite complex processing pipelines, iterating through many different steps until reaching the final output. One example for such an experiment is ptychography [13–15], which enables one to build up very large images by combining the large field of view of a high-precision scanning microscope system with the resolution provided by diffraction measurements.

Ptychography uses a small step size relative to the size of the illuminating beam when scanning the sample, continuously generating large redundant datasets that can be reduced into a high-resolution image. Resolution of a ptychography image does not depend directly on the size or shape of the illumination. X-ray wavelengths can probe atomic and subatomic scales, although resolution in scattering experiments is limitated by other factors such as radiation damage, exposure, and brightness of the source to a few nanometers except in special cases (such as periodic crystals). To reconstruct an image of the object from a series of X-ray scattering experiments, one needs to solve a difficult phase retrieval problem, because at short wavelengths it is only possible to measure the intensity of the photons on a detector. The phase retrieval problem is made tractable in ptychography by recording multiple diffraction patterns from overlapping regions of the object, providing redundant datasets to compensate for the lack of the phase information. The problem is made even more challenging in the presence of noise, experimental uncertainties, optical aberrations, and perturbations of the experimental geometry which require specialized solvers and software [16–18].

In addition to its reconstruction pipeline, a ptychography experiment involves additional I/O operations such as calibrating the detector, filtering raw data, and communicating parameters (such as X-ray wavelength, scan positions, detector distance, and flux or exposure times) to the analysis infrastructure.

Large community driven projects have developed frameworks optimized for distributed data stream processing. Map-reduce-based solutions such as Hadoop [19, 20] and Spark [21] provide distributed I/O, a unified environment, and hooks for running map and reduce operations over a cloud-based network. Other frameworks such as Flink [22], Samza [23], and Storm [24] are more tailored for real-time stream processing of tasks executing a directed acyclic graph (DAG) [25] of operations as fast as possible. Workflow graphs such a Luigi [26] and Dask distributed [27, 28] provide an iterative component, but are either optimized for batch processing and workers are treated as a singular entity able to execute the DAG in its entirety.

Such frameworks target operations as a unit of tasks and generalize the notion of resources; however, the ecosystem is harder to decentralize. These paradigms are not easily mappable to a production beamline environment, where processing algorithms working on data from a detector might be running on a field-programmable gate array (FPGA), the motion control system on a real-time MCU, the acquisition control on a windows operating system, and the scientist a macOS laptop. The rest of the pipeline tasks might hop to several different architectures including CPUs for latency bound tasks, and GPUs for high-throughput image processing and visualization. While frameworks such as Flink along with Kafka [29] (high-throughput distributed message system) and ZooKeeper [30] (distributed coordination and management) can be adopted to fit the described processing environment, our solution at a lower level accomplishes the same task with less computational and human resources.

Nanosurveyor is a modular framework to support distributed real-time analysis and visualization of data. The framework makes use of a modular infrastructure similar to Hummingbird [31] developed to monitor flash X-ray imaging experiments at free electron lasers (FELs) with high data rates in real time over multiple cores and nodes. Similar frameworks and pipelines have been also implemented in other research fields such as serial crystallography [32], cryo-electron microscopy [33], or functional magnetic resonance imaging [34].

Within this framework, we developed a streamlined processing pipeline for ptychography which unifies all components involved and allows scientists to monitor and quickly act upon changes along the experimental and computational pipeline.

## Methods: real-time streaming framework

Nanosurveyor was developed to provide real-time feedback through analysis and visualization for experiments performed at synchrotron facilities, and execute a complex set of operations within a production environment. Its design is such that it can be effectively adapted to different beamline environments. It is built around a client–server infrastructure allowing scientists to use facility resources while located at a beamline or remotely, operating on live data streamed from the beamline. Additionally, one can use the Nanosurveyor user interface for off-line processing of experimental data saved on disk. In this section, we describe the resources and capabilities provided by the modular streaming infrastructure.

### Terminology primer

As the streaming pipeline architecture is heavily dependent on a variety of resources which uses terminology more common in computational sciences, a brief primer is necessary to ensure both completeness and clarity for the rest of the paper.

- User interface: The user interface describes the visual layer and provides the interaction between user and hardware. PyQt [35] and PyQtGraph [36] serve as the core interface and visualization layer as these are popular graphics libraries which allow for easy customization to serve processing needs.
- Communication: To ensure generality of the modular components within Nanosurveyor, communication between modules and throughout the system is important. ZeroMQ, a communication interface [37], allows the internal architecture to communicate using common communication patterns while ensuring data are queued for processing and delivered to destination successfully. A core component of the communication interface is sockets acting as plugs between different modules.
- Event loop: Actions driven by events are at the core of Nanosurveyor. This design allows for immediate response critical to a streaming pipeline. When an event such as reading occurs, the core pipeline pushes the information or metadata to the appropriate events waiting in the pipeline. These actions take place until no other actions need to be performed.

### Modular framework

**Fig. 1:**
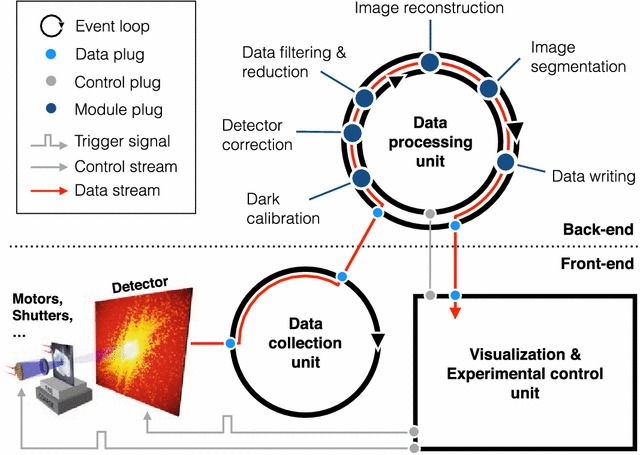
Streaming pipeline: Overview of the real-time streaming framework of Nanosurveyor. The modular server–client infrastructure is divided into a back-end (running the data processing unit) and a front-end (running the visualization and control unit). The data flow is depicted as a *red arrow*, while communication channels for controlling experiment and back-end are shown in *gray*. Once an experiment has started (trigger signal), the data collection unit continuously receives new data packets from a detector and sends raw data frames to the data processing unit. Depending on the specific needs of the experiment, different modules (from dark calibration to data writing) can be plugged into the pipeline. At all times, there is an active connection (asynchronous socket communication) between all components (including the visualization interface) allowing the scientist to monitor progress while data are still being acquired and processed

As described above, Nanosurveyor is designed to be adaptable and modular. Therefore, we designed it with a client–server infrastructure (Fig. 1) enabling scientists to run their experiment while at the beamline or remotely from their institution. This strategy also allows the client to be very light and flexible while the server can be scaled according to the resources needed.

The Nanosurveyor infrastructure equips each module with two fundamental capabilities. First, a description format language of key-value pairs allows every module to describe its input and output. Second, it provides the ability to describe the connection between the modules, including the front-end.

The capability to connect the communication path between modules allows the end-to-end pipeline to be constructed and described seamlessly. This is done through a proxy communication layer allowing the modules to run either closely together or on completely separate machines. This strategy is transparent to the beamline user and accommodates both environments with centralized resources as well as those where resources are spread across a network.

Additionally, as each module in the pipeline can be executed in its own environment, Nanosurveyor provides dynamic parallelism by allowing the user to scale the number of resources available to each step: this is done by treating each stage as a worker process that can be scaled up or down to address bottleneck or performance issues.

### Software stack

The core components of the Nanosurveyor streaming software are written in Python using ZeroMQ, a high-performance messaging library [37] for network communication, PyQt4 [35] and PyQtGraph [36] for the graphical user interface (GUI) and visualization, and Numpy [38] together with Scipy [39] for manipulation of data arrays. For some components, we used C extensions in order to boost the performance to meet the demands of producing a real-time interactive tool running at the beamline. With all these dependencies pre-installed, Nanosurveyor can be simply installed in the standard Python way: python setup.py install.

Python is a language with a robust and active community with libraries that are well tested, supported, and maintained. Additionally, the choice of Python allows our infrastructure to be flexible to the demands of varying requirements of different processing pipelines. The ptychography pipeline (discussed in detail later in the paper) contains GPU optimized code and Python binding support easily allows the Nanosurveyor infrastructure to provide support for these types of hybrid architectures. The framework currently runs on Mac, Linux, and Linux-based cluster environments, and can be extended to Windows platforms depending on support for module dependencies. The core components that Nanosurveyor depends on are available on all major platforms.

### Communication

A critical component in generating usable real-time pipelines relies on the communication infrastructure. This enables a clear and concise separation of the inputs and outputs at the module level. Furthermore, it defines how modules communicate from beginning to end, and ensures that tasks are load-balanced to achieve the appropriate performance characteristics of the pipeline.

The communication in Nanosurveyor uses Javascript Object Notation (JSON) [40], an industry standard way of conveying metadata between modules as well as between the front-end and back-end. The metadata provides a human readable component.

ZeroMQ provides the communication backbone of the Nanosurveyor infrastructure. Using the publisher–subscriber model for the core components enables Nanosurveyor to provide a load-balancing scheme, which uses a backlog queue to avoid losing data when sufficient resources are not be available. The execution pipeline creates a command port and a data port. The command port allows metadata to reach and update parameters as well as return responses to keep status requests alive and provide feedback on the current state of the running module. The data port moves data through the pipeline, running the actionable item within each module and moving the result to the output queue to be processed by the next stage of the pipeline.

Two types of configurations are required: front-end and back-end. The front-end sets up the variables necessary for each module to function while the back-end configuration is responsible for allocating resources, balancing the load of workers, scheduling activities, and communicating between modules while providing feedback to the front-end.

These two components provide the Nanosurveyor infrastructure with the information it needs to establish the relevant connections, receive and send parameters to ensure proper configuration, and introspect the state of parameters and data to provide visual feedback to the user when running through the processing pipeline.

### Client–server architecture

The Nanosurveyor framework consists of an assortment of core components that ensure that the front-end provides easy to use and adaptable interface while the back-end is efficient, resilient, and responsive. The individual processing modules are all based on the same structure: an event loop runs routing data from the control and data sockets, waiting for tasks, asking the handler for configuration parameters (JSON string), and processing data (receiving/sending through the data socket).

#### Back-end

The main back-end handler is running a big ZeroMQ event loop. The main task of the handler is to register the modules that run on the back-end and ensure data and control paths are appropriately connected up and running. It also does the following:

- Launches all the processing modules as separate processes (single-core or MPI) and keeps track of the jobs started. This can be done with a batch processing system such as SLURM (or any other queuing system) or by launching separate python processes;
- Creates the sockets for streaming pipeline, which is a list of control and data sockets communicating between the handler and all the processing modules as well as the data collector and the interface;
- Runs the event loop, takes commands, deals out data packets, and handles everything in the back-end including user interruption and other control and configuration commands.

#### Data tracking

Tracking and ensuring the correctness of data is an important part of the execution pipeline. The Nanosurveyor framework provides a module called nscxwrite which allows customized writing of files at different stages of the data acquisition pipeline (raw, filtered, and reconstructed). This capability provides several benefits, such as assurances to users that data move correctly from module to module and are not corrupted along the way, as well as an ability to debug an algorithm that is executed within a complex sequence of events.

Furthermore, the ability to save intermediate data can be enabled or disabled (for performance reasons or to reduce storage) as well as customized. The framework also comes with a standalone script called nsraw2cxi, that translates raw detector data to processed CXI files, and a script to stream simulated FCCD data through the pipeline for testing. The data format of the output files follows the CXI file format [41].

#### Logging

Nanosurveyor also provides a way to debug a complex pipeline through logging of both the output and error channels which includes communication between modules as well as output and error that arise from within modules.

The output of all modules is piped to STDOUT and STDERR within the file system running each process ($HOME/.nanosurveyor/streaming/log/).

This is a useful tool that invokes tail -f on the piped out/err files, making it possible to monitor what is going within the individual processing modules.

### Graphical user interface

For the front-end, the framework provides a versatile GUI based on PyQt4 and PyQtgraph for monitoring, visualizing, and controlling the data processed live or post-processed through the pipeline. PyQt4 (built on Qt) provides the ability to construct and modify the user interface to easily add and remove functionality while PyQtgraph provides access to advanced visualization functionality for data that can be represented as images or volumes. Several common operations provided through the framework include the following:

- View the content of already processed files: inspect reconstructions from collected data and provide other useful utilities (histograms, error line plots, correlation plots, and others);
- Control and monitor the streaming: configure streaming, inspect live reconstruction, monitor performance (upload/download rates, status update of the streaming components);
- Simulate an experiment starting from an SEM image or similar;
- Process and inspect, through a provided interface, data from custom modules processed on the back-end (e.g., data from a ptychography or Tomography reconstruction).

Generally speaking, the design facilitates adding new modules to the GUI, e.g., a viewer for tomograms or similar. This flexibility allows the front-end to be customized for different beamline processing environments.

Finally, the architecture aims to be modular in the front- and back-end of the client–server architecture, meaning that there is a template structure for the basic features of a processing module. Additionally, in principle, any given processing module can be hooked into this network (e.g., tomography, spectral analysis, or any other image analysis).

## Results: streaming ptychography

**Fig. 2:**
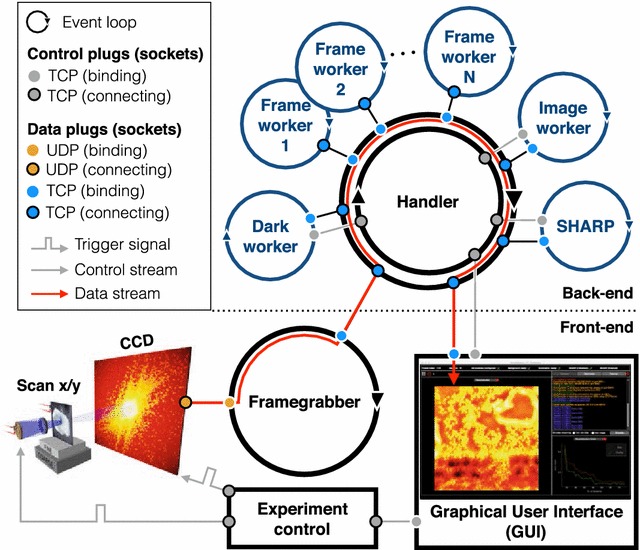
Ptychographic streaming pipeline: streaming pipeline implemented at the ALS for ptychographic imaging. The software structure follows the same logic as sketched in Fig. 1. Once a new scan has been triggered by the experimental control, a frame-grabber continuously receives raw data packets from the camera, assembles them to a frame and sends raw frames to the back-end. Incoming frames are processed by different (and independent) workers of the back-end and reduced data are sent back to the front-end and visualized in a graphical user interface (GUI). The pipeline includes a dark worker for dark correction, multiple frame workers for pre-processing and data reduction, an image worker for low-resolution image reconstruction and a SHARP worker for high-resolution ptychographic image reconstruction. A handler is coordinating the data and communication workflow in which different types of control and data plugs (sockets) are used. While most of the components communicate via the transmission control protocol (TCP), the raw data packets from the camera are sent via the user datagram protocol (UDP)

We adapted the outlined streaming framework described above for the specific needs of ptychography and are currently implementing this ptychography streaming pipeline at the beamline for scanning transmission X-ray microscopy (STXM) at the ALS. The main motivation for this project is to make high-resolution ptychographic reconstructions available to the scientist in real-time. To achieve this goal, we streamlined all relevant processing components of ptychography into a single unit. A detailed outline of our pipeline is sketched in Fig. 2.

As described in the previous sections, we follow the idea of a modular streaming network using a client–server architecture, with a back-end for ptychographic processing pipeline and a front-end for configuration, control and visualization purposes.

On the back-end side, the streaming infrastructure is composed of a communication handler and four different kinds of workers addressing dark frames, diffraction frames, reduced and downsampled images and the ptychographic reconstruction using a software package for scalable heterogeneous adaptive real-time ptychography (SHARP [18]). The handler bridges the back-end with the front-end and controls the communication and data flow among the different back-end workers. The dark worker accumulates dark frames and provides statistical maps (mean and variance) of the noise structure on the detector. The frame workers transform raw into clean (pre-processed) diffraction frames. This involves a subtraction of the average dark, filtering, photon counting, and downsampling. Depending on the computing capacities of the back-end, it is possible to run as many frame workers simultaneously as needed. The image worker reduces a collection of clean diffraction frames, producing low-resolution image reconstructions and an initial estimate of the illumination function which, together with the clean diffraction frames, is then feeded as an input for the high-resolution ptychographic reconstruction worker (SHARP).

**Fig. 3:**
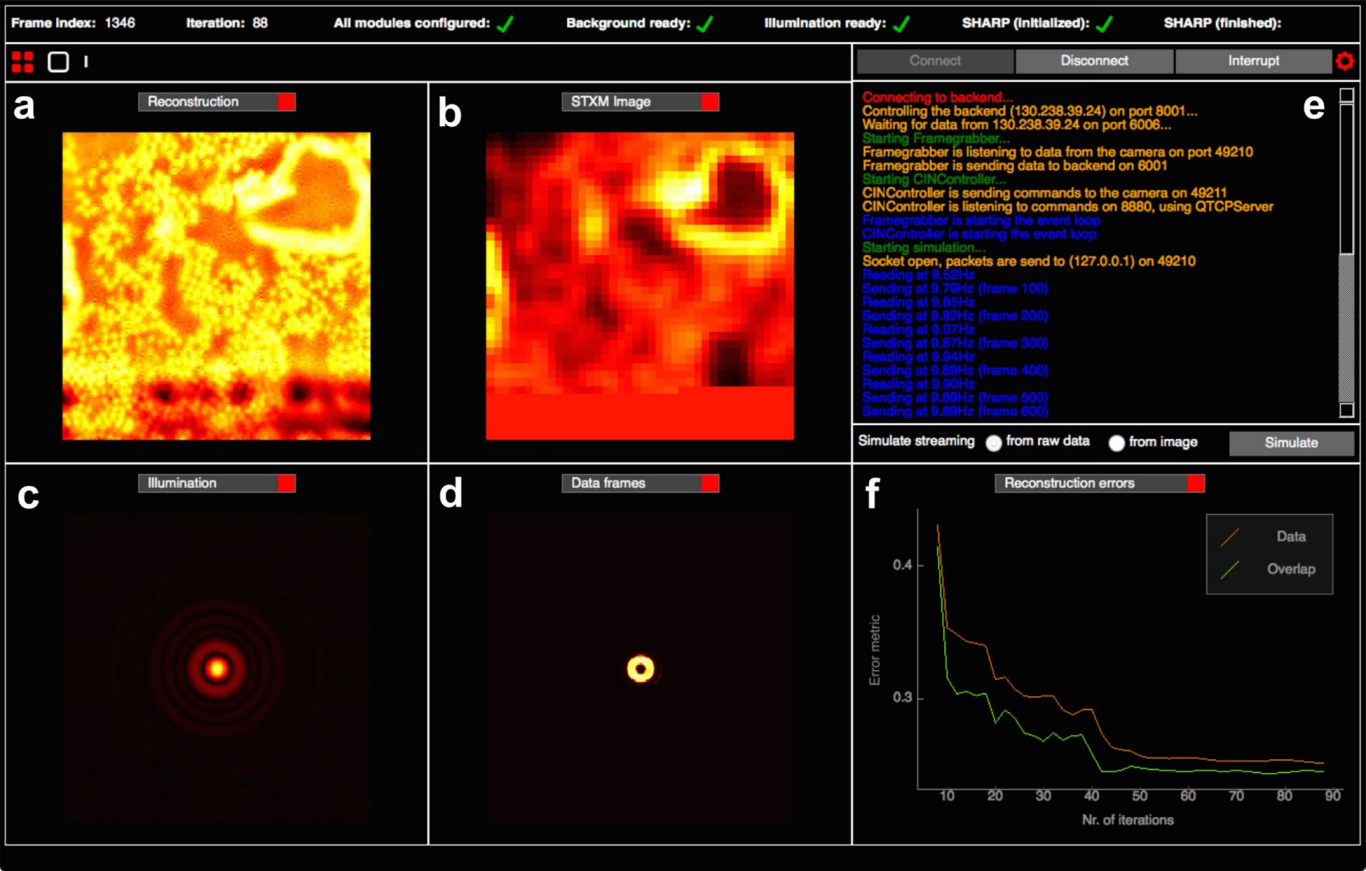
Graphical user interface (GUI): for the ptychographic streaming pipeline implemented at the ALS. The interface provides **a** real-time view of the ptychographic reconstruction (high resolution), **b** real-time view of the STXM analysis (low resolution), **c** current guess of the illumination function, **d** current processed data frame, **e** logging and error messages and **f** error metrics of the iterative reconstruction process, and other control and monitoring elements around

The front-end consists of a worker that reads raw data frames from a fast charge-coupled device (FCCD) [42], coordinating with a separately developed interface for controlling the experiment (such as motors and shutters) and a graphical user interface (GUI) which is used both for visualizing and controlling the ongoing reconstruction. An example view of the GUI for streaming ptychography is shown in Fig. 3.

Following the data flow along the streaming pipeline, the starting trigger comes from the control interface which initiates a new ptychographic scan providing information about the scan (step size, scan pattern, number of scan points) and other relevant information (e.g., wavelength) to the back-end handler. Simultaneously, the control sends triggers to the scanning motors and the FCCD. A typical ptychographic scan combines the accumulation of a given number of dark frames together with scanning the sample in a region of interest. The frame-grabber, already waiting for raw data packets to arrive, assembles the data and sends it frame-by-frame to the back-end handler. When dealing with an acquisition control system that runs independently, the handler can distinguish between dark and data frames using counters. Dark and data frames are distributed to the corresponding workers. Having clean diffraction frames and an initial guess for the illumination ready, the SHARP worker is able to start the iterative reconstruction process. SHARP initializes and allocates space to hold all frames in a scan, computes a decomposition scheme, initializes the image and starts the reconstruction process. Unmeasured frames are either set to a bright-field frame (measured by removing the sample) or their weight is set to 0 until real data are received.

Depending on the configuration, data at different states within the streaming flow can be displayed in the GUI and/or saved to a CXI file via the nscxiwrite worker module.

All components of the streaming interface run independent event loops and use asynchronous (non-blocking) socket communication. To maximize performance, the front-end operates very close to the actual experiment, while the back-end runs remotely on a powerful GPU/CPU cluster.

### Pre-processing of FCCD data

We developed the following processing scheme for denoising and cleaning the raw data from the FCCD and preparing frames for the ptychographic reconstruction,

1. Define center (acquire some diffraction frames and compute the center of mass if needed). This is needed for cropping, and to deal with beamstop transmission;
2. Average dark frames: we first acquire a sequence of frames when no light is present, and compute the average and standard deviation of each pixel and readout block. We set a binary threshold to define bad (noisy) pixels or bad (noisy) ADC channels, when the standard deviation is above a threshold or if the standard deviation is equal to 0;
3. Remove offset using the overscan linear or quadratic offset: stretch out the readout sequence in time and fit a second order polynomial over the overscan;
4. Identify the background by thresholding;
5. Perform a Fourier transform of the readout sequence of the background for each channel, remove high frequency spikes by thresholding, and subtract from data;
6. Threshold signal below 1 photon;
7. Divide by beamstop transmission;
8. Crop image around center;
9. Downsample: take a fast Fourier transform (FFT), crop or multiply by a kernel (e.g., Gaussian) and take inverse fast Fourier transform (IFFT).

### Simulation

For testing the functionality and performance of the streaming ptychography pipeline as well as exploring different configurations, we developed a protocol that simulates an entire ptychography scan. Using a simulated illumination from a Fresnel Zone Plate (FZP) and basic scan parameters (number of scan points, the scanning step size, the scanning pattern), diffraction patterns from a well-known test sample are calculated in the same raw data format as those generated by the FCCD. As a last step, Poisson noise and a real background are added to the data. These raw data packets together with the simulated metadata are introduced to the end-to-end streaming pipeline and produce outputs as shown in Fig. 3.

One major benefit of this feature is the ability to scale and test the pipeline at different acquisition rates and therefore be able to provide performance metrics on the behavior of a sequence of algorithms enabling developers to further improve their execution pipeline.

In a simple performance test, we simulated a 40 × 40 scan producing 1600 raw data frames which were sent by a virtual FCCD at a rate of 10 Hz. At the end of the pipeline, we observed a complete reconstructed image after around 5 min. This translates into a streamlining pipeline rate of about 2 Hz, with most of the time spent on filtering and cleaning the individual frames. A significant portion of the pre-processing time is unique to the FCCD pipeline. While this rate is still far from ideal, it can be easily be sped up and scaled by using parallel execution, load-balancing strategies, and eventually through high-throughput GPU optimizations. With further improvements on the performance on the individual components as well as optimization of the network communication, we expect a substantial increase of the processing rate.

### Experimental data

Experimental data produced by the FCCD can involve missing frames, corrupted frames, and timing issues between different hardware and software components. In addition, the correct choice parameter values for the ptychographic reconstruction might be inherent to the data itself and can thus carry from experiment to experiment. To make Nanosurveyor more robust for such cases, it is desirable to expose configuration parameters as a runtime or heuristic feature rather than determined them at execution time, and take a more data-based approach where options are set based on feature detection.

## Discussion

Performance considerations and additional limitations must be understood and considered in integrating such an execution pipeline in a production environment. While the following list is not comprehensive, in building this environment, we have considered the following:

- Limits (performance, algorithm, memory, disk) to software and hardware need to be considered. The Nanosurveyor infrastructure provides logging support while the ZeroMQ publisher–subscriber model allows a stuck or crashed process to be replaced with another. The current solution Nanosurveyor can be made more robust and this is work that is considered as active and ongoing;
- Hardware failures are inevitable in a production environment involving machinery. Recovery from these types of issues requires customization for each beamline environment. Within Nanosurveyor, there is a heartbeat for each module and a base mechanism within the framework to inform the user that a failure (or multiple failures) might have occurred;
- Interrupting experiments should be a core use case of any real-time feedback loop when trying to get an understanding of the data as quickly as possible. Once information about the material is flowing through the computational pipeline, it is valuable to be able to determine if an experiment is, in fact, failing or uninteresting. This can occur in many ways such as wrong setup, wrong material, or wrong region of scanning. For these scenarios, it is prudent for a working pipeline to be able to abort, clear out the pipeline, and reset itself;
- Expensive operations and algorithms executed in a beamline operating environment may have varying degrees of performance characteristics. These characteristics can often slow down the overall pipeline if any one of the operations is inefficient. Nanosurveyor attempts to get around this issue in two ways: first, it allows for a load-balancing approach where more workers can be added to the expensive stages of the pipeline. Second, using the ZeroMQ queue, the beamline can still operate with the slowdown and backlog while ensuring that the pipeline can continue to function, at least until hardware memory runs out. This issue can also be mitigated by evaluating the performance of the module and if possible optimizing the algorithm as well;
- As data rates might increase both in speed and size, we have several optimization strategies, including parallelizing computation load across a bigger back-end, speeding up expensive algorithms, and extracting or identifying important information earlier to reduce size and complexity.
- For current 2D and 3D visualization requirements, it is sufficient to use PyQtGraph. However, if the data volume gets large enough such that it would be difficult to view raw output, visualization frameworks supporting distributed rendering strategies could be used. More advanced approaches such as adaptive downsampling, compression, or other visualization representations could be swapped into the pipeline if needed.

## Conclusions and future work

This work introduced Nanosurveyor—a framework for real-time processing at synchrotron facilities. The infrastructure provides a modular framework, support for load-balancing operations, the ability to run in a distributed client–server mode, and gives feedback on each stage of a complex pipeline.

The framework was adapted to support streamlined pipelines for ptychography. In this case, expensive stages such as pre-processing are load-balanced with multiple workers, and image reconstruction are parallelized over MPI to compute efficiently in a distributed manner. Results from every stage of the pipeline are then transmitted to the front-end, providing users at the beamline comprehensive knowledge of the experiment and of how the data are transformed from start of acquisition to end output. Although the Nanosurveyor framework provides several core capabilities that are necessary for operating at typical beamlines, there are several key advances that we are currently working on to make the computational pipeline complete. A couple of highlights include the following:

*Iterative execution, instrument control* Adding support for controlling the beamline itself will complete the current pipeline and provide an iterative execution loop enabling future pipelines to adaptively acquire and analyze data from the operating beamline, and automatically request more data when necessary. For example, if the reconstruction detects bad frames, or that the sample has drifted, then more frames can be automatically requested on the fly without interrupting the overall experiment. If the reconstruction determines that part of the image being acquired is empty or uninteresting it could request fewer frames and focus on the relevant part of the sample.

*Optimizing pipeline execution* Currently, communication occurs over ZeroMQ providing many benefits, including dealing with backlog, automated load-balancing, and the ability to interleave work running different stages of the execution pipeline. We are also investigating ways to fuse modules to optimize execution times. Making communication agnostic by using handles enables efficient use of memory optimization strategies, socket communication, or saving on data movement costs, e.g., transferring data between GPU-based modules by moving a pointer rather than copying data.

In conclusion, we have presented a framework that is built to run at modern beamlines, can handle the geographic considerations between users and experiments running at synchrotron facilities, and supports real-time feedback. These features, along with the modular design, provide a foundation that can be extended and readily deployed on many of the beamlines in use today.
